# New chemosynthetic route to linear ε-poly-lysine†
†Electronic supplementary information (ESI) available. See DOI: 10.1039/c5sc02479j


**DOI:** 10.1039/c5sc02479j

**Published:** 2015-07-28

**Authors:** Youhua Tao, Xiaoyu Chen, Fan Jia, Shixue Wang, Chunsheng Xiao, Fengchao Cui, Yunqi Li, Zheng Bian, Xuesi Chen, Xianhong Wang

**Affiliations:** a Key Laboratory of Polymer Ecomaterials , Changchun Institute of Applied Chemistry , Chinese Academy of Sciences , Renmin Street 5625 , 130022 , People's Republic of China . Email: youhua.tao@ciac.ac.cn ; Email: xhwang@ciac.ac.cn ; http://www.youhuatao.weebly.com/

## Abstract

ε-Poly-lysine (ε-PL) is an uncommon cationic, naturally-occurring homopolymer produced by the fermentation process. Due to its significant antimicrobial activity and nontoxicity to humans, ε-PL is now industrially produced as an additive, *e.g.* for food and cosmetics. However, the biosynthetic route can only make polymers with a molecular weight of about 3 kDa. Here, we report a new chemical strategy based on ring-opening polymerization (ROP) to obtain ε-PL from lysine. The 2,5-dimethylpyrrole protected α-amino-ε-caprolactam monomer was prepared through cyclization of lysine followed by protection. ROP of this monomer, followed by the removal of the protecting group, 2,5-dimethylpyrrole, ultimately yielded ε-PL with varying molecular weights. The structure of this chemosynthetic ε-PL has been fully characterized by ^1^H NMR, ^13^C NMR, and MALDI-TOF MS analyses. This chemosynthetic ε-PL exhibited a similar p*K*_a_ value and low cytotoxicity as the biosynthetic analogue. Using this new chemical strategy involving ROP without the need for phosgene may enable a more cost effective production of ε-PL on a larger-scale, facilitating the design of more advanced biomaterials.

## Abstract 2

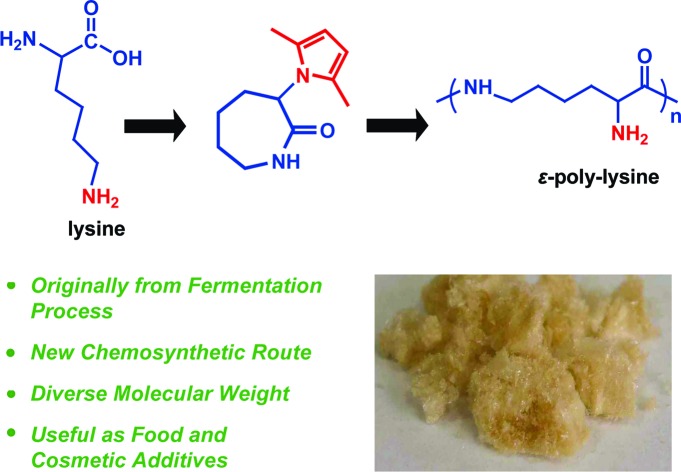
ε-poly-lysine (ε-PL) is a naturally-occurring homopolymer produced by the fermentation process. Here, we report a new chemical strategy based on ring opening polymerization to obtain ε-PL from lysine.

## Introduction

Lysine is a naturally available renewable resource, and recent advances in aerobic fermentation have made it possible to obtain lysine more efficiently, with both high purity and at a low price.^1^ The homopolymer of lysine incorporates a variety of favorable features, particularly due to its large number of reactive amino groups and the resulting polycationic characteristic, chirality, and biodegradability.^2^–^9^ Therefore, poly(lysine) is now widely used in chemical, pharmaceutical, food, biotechnology and other industries.^10^–^23^

**Scheme 1:**
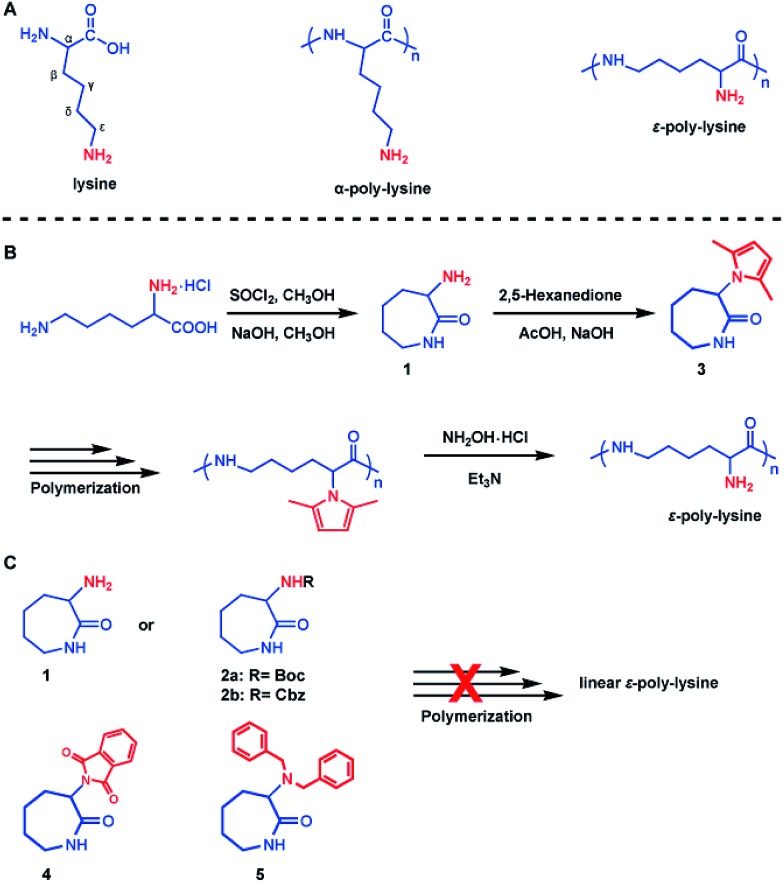
Synthesis of ε-poly-lysine. (A) Illustration of the chemical structure of lysine, α-poly-lysine and ε-poly-lysine. (B) The concept of the chemical approach for the preparation of ε-poly-lysine. (C) Designed monomers for possible polymerization.

As shown in Scheme 1A, lysine contains two amino groups. Therefore, two types of linear homopolymer structures are possible depending on the linkage mode between the amino groups and carboxyl groups in lysine.^24^–^27^ Ring-opening polymerization of lysine *via* the *N*-carboxyanhydride (NCA) intermediate has already been shown to be a feasible approach in the production of α-poly-lysine (α-PL).^28^–^30^ However, this process requires the use of highly toxic phosgene gas or phosgene analogues, strict water removal, and high monomer purity. Furthermore, α-PL exhibits cytotoxic effects, limiting its use in biomedical applications.^31^–^36^ ε-Poly-lysine (ε-PL), bearing α-amino groups on the side chains, is a naturally-occurring homopolymer, containing an amide linkage between the ε-amino groups and α-carboxyl groups of lysine. The material was accidentally discovered in 1977 as an extracellular material produced by enzymatic fermentation of *Streptomyces albulus*.^37^ In contrast to α-PL, ε-PL is edible and nontoxic for humans.^38^,^39^ Moreover, it shows remarkable stability towards high temperatures as well as alkaline or acidic conditions, and it shows significant antimicrobial activity.^40^ Due to these features, linear ε-PL is produced on an industrial scale by a fermentation process and it has a variety of applications, *e.g.* as an additive in food and cosmetics, biodegradable fibers, highly water absorbable hydrogels and drug carriers.^38^,^41^ However, the biosynthetic route yields the target material with a molecular weight of only about 3 kDa, and no copolymers composed of lysine and other amino acids can be found in the producer strains, probably because the two amino acids are polymerized by different enzymes.^40^ Consequently, the composition and properties of the resultant polymers are hard to regulate.

Synthetic chemistry can be used to prepare macromolecules with diverse chemical structures. Moreover, in comparison to biosynthetic technologies, chemical procedures may enable a more cost-efficient production of ε-PL on a larger-scale. Unfortunately, fully synthetic protocols are more challenging than biotechnological pathways, and are limited to oligomer synthesis. The most obvious chemical method for preparing oligomers of ε-PL is to use solid-phase synthesis.^40^,^42^ However, this route is extremely tedious, requiring repetitive protection/deprotection reactions in which one amino acid at a time is added to elongate the chain. Hence, a more effective chemical polymerization of lysine into linear ε-PL with varying molecular weights is needed. Ring-opening polymerization (ROP) is one of the most facile and commonly used methods for the synthesis of polymers. However, due to the synthetic difficulties encountered with large cyclic monomers (nine-membered *N*-carboxyl-ε-amino acid anhydride, ε-NCA) of l-lysine,^43^ the chemosynthesis of ε-PL *via* ROP has never been reported until now.

We herein present a conceptually novel approach for the preparation of ε-PL with varying molecular weights. We hypothesized that the synthesis of the seven-membered lactam monomers from lysine, and ROP of such monomers would produce ε-PL. In the present report, a 2,5-dimethylpyrrole protected α-amino-ε-caprolactam (MPCL) monomer **3** was first prepared through cyclization of lysine followed by protection (Scheme 1B). The linear ε-PL was subsequently produced by ROP of the MPCL monomers, followed by the removal of the 2,5-dimethylpyrrole protecting group. The chemosynthesis of ε-PL *via* ROP can produce ε-PL with diverse molecular weights and chemical compositions, which, to the best of our knowledge, has never been reported before. These chemosynthetic ε-PLs offer highly promising properties for a variety of applications, *e.g.* as a cosmetic additive or in biodegradable products.

## Results and discussion

### α-Amino-ε-caprolactam monomer

The attempt to synthesize the seven-membered lactam monomer from lysine and polymerize it to make linear ε-PL has attracted our interest. Initially, we prepared α-amino-ε-caprolactam monomer **1** that may result in ε-PL *via* ROP. Commercially available l-lysine monohydrochloride was used as a starting material and the α-amino-ε-caprolactam monomer was prepared *via* esterification of l-lysine monohydrochloride with methanol in thionyl chloride, followed by cyclization in the presence of sodium hydroxide (Scheme 1B). Acylation of **1** with Boc or Cbz protecting groups gave lactams **2a** and **2b**. The structures of the monomers were characterized by ^1^H NMR spectroscopy, ^13^C NMR spectroscopy and ESI-MS (Fig. S1–S5†). Sodium was used for the ROP of these lactam monomers because sodium is a “green” metal and its residue in products will not hamper the final applications. However, only crosslinked products with both α-linear and ε-linear linkages were obtained, indicating that the NH_2_-unprotected lactam **1** or monomers with common protecting groups, such as Boc (**2a**) and Cbz (**2b**), could not be polymerized efficiently to produce linear ε-PL. For lactam **1**, both α-amino and ε-amide could be deprotonated in the presence of sodium. “Activated monomers” with double propagation active species were generated *in situ*. For lactams **2a** and **2b**, proton abstractions from the α- and ε-amide also produced “activated monomers” with double propagation active species.^44^ Therefore, an alternative protecting strategy was adopted to produce the desired ε-PL.

### 2,5-Dimethylpyrrole protected α-amino-ε-caprolactam monomer

Our synthesis approach for linear ε-PL focused on the choice of protecting groups, since we believe the α-amino group interferes with the crucial polymerization step. The following characteristics are key for an appropriate amino-protecting group: (a) dual protection of amino functions; (b) less steric hindrance; (c) thermo-stability during polymerization; (d) good solubility of the protected monomers and polymers in organic solvents; and (e) ability to be removed under mild conditions. The listed requirements precluded the use of dibenzyl or phthalimide protecting groups (Scheme 1C and Fig. S7†) due to their large steric hindrance, thus greatly reducing the arsenal of potential protecting groups. Of the remaining possibilities, 2,5-dimethylpyrrole seems to fit our protecting group requirements.^45^,^46^ This rarely used amino-protecting group indeed proved to be the right choice for our purpose as its use resulted in the successful synthesis of the desired linear ε-PL.

**Fig. 1:**
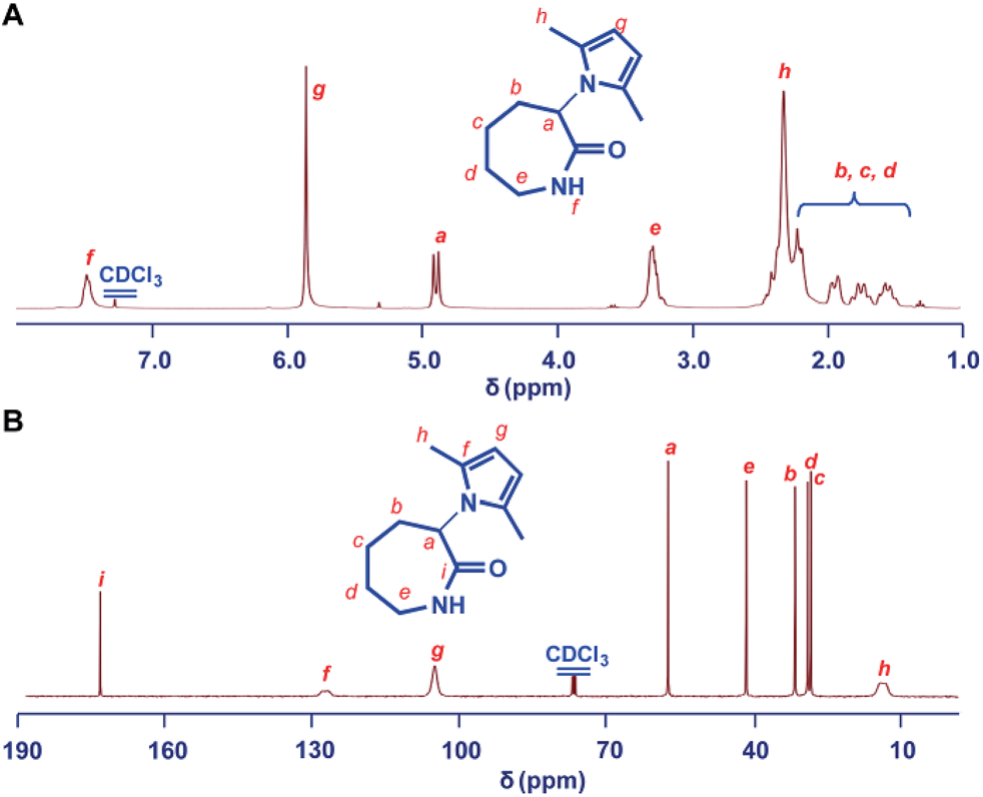
^1^H NMR (A) and ^13^C NMR (B) spectra of the 2,5-dimethylpyrrole protected α-amino-ε-caprolactam (MPCL) monomer. All spectra were measured in CDCl_3_ at room temperature.

The 2,5-dimethylpyrrole protected α-amino-ε-caprolactam (abbreviated as MPCL) **3** was synthesized by reacting the amine group in lactam **1** with 2,5-hexadiione (Scheme 1B). The crude product was purified by column chromatography and characterized by ^1^H NMR (Fig. 1),^13^C NMR (Fig. 1) and ESI-MS analyses (Fig. S6†).

This MPCL monomer **3** was subsequently polymerized using sodium as a catalyst. The resulting polymer of MPCL (PMPCL) was a hard, tough and light-colored mass. As shown in Table 1, the yield of the polymerization at 260 °C was in the range of 62–85%, corresponding to Na/MPCL feed ratios varying from 10 to 0.5%. The molecular weights and PDIs of the obtained PMPCLs were characterized by gel permeation chromatography (GPC) and are shown in Table 1. The *M*_n_ of the polymer was increased dramatically by decreasing the weight ratio of Na to MPCL. It was only 2.5 kDa when the ratio of Na to MPCL monomer was 10% and increased about 5-fold to 17.1 kDa at 1% catalyst loading. It was further raised about 1.5 times to 26.1 kDa at 0.5% catalyst loading. Polymerizations at 240 °C did also result in the formation of polymers. However, the corresponding yields were found to be lower, and solidification was observed to occur at a later point (Table 1, entry 6). Presumably, the lower viscosities at higher temperatures allow for higher mobility of the growing chains in the polymerization mass and consequently lead to higher conversions. The optimization of the polymerization time was then explored. It was observed that the *M*_n_ of the polymer was about 50 kDa when the polymerization time was 1.5 hours at 0.5% catalyst loading (Table 1, entry 9). It was also found that the *M*_n_ of the obtained PMPCL decreased with prolonged polymerization time at 260 °C (Table 1, entries 1 and 9). We believe that this change in *M*_n_ indicates a change in the chain-length of PMPCL. Such a shift could occur readily on continued heating at elevated temperatures by amide interchange under the influence of the ion or salt that initiated the reaction.

**Table 1:** Bulk ring-opening polymerization of MPCL **3** catalyzed by sodium^*a*^

Entry	Na : MPCL **3**^*b*^ (%)	Time (h)	Temp. (° C)	Yield^*c*^ (%)	*M*_n_ ^*d*^ (kDa)	*M*_w_/*M*_n_^*d*^
1	0.5	2	260	62	26.1	1.8
2	1	2	260	69	17.1	1.8
3	2	2	260	78	13.8	1.8
4	5	2	260	76	7.5	1.7
5	10	2	260	85	2.5	1.4
6	1	2	240	50	16.2	1.6
7	0.5	0.5	260	40	27.1	1.5
8	0.5	1	260	55	33.8	1.6
9	0.5	1.5	260	58	49.2	1.6

^*a*^The reaction was performed in bulk in a 10 mL flame-dried polymerization tube.

^*b*^Weight percent.

^*c*^Isolated yield.

^*d*^The number-average molecular weight (*M*_n_) and distribution (*M*_w_/*M*_n_) were determined by GPC.

**Fig. 2:**
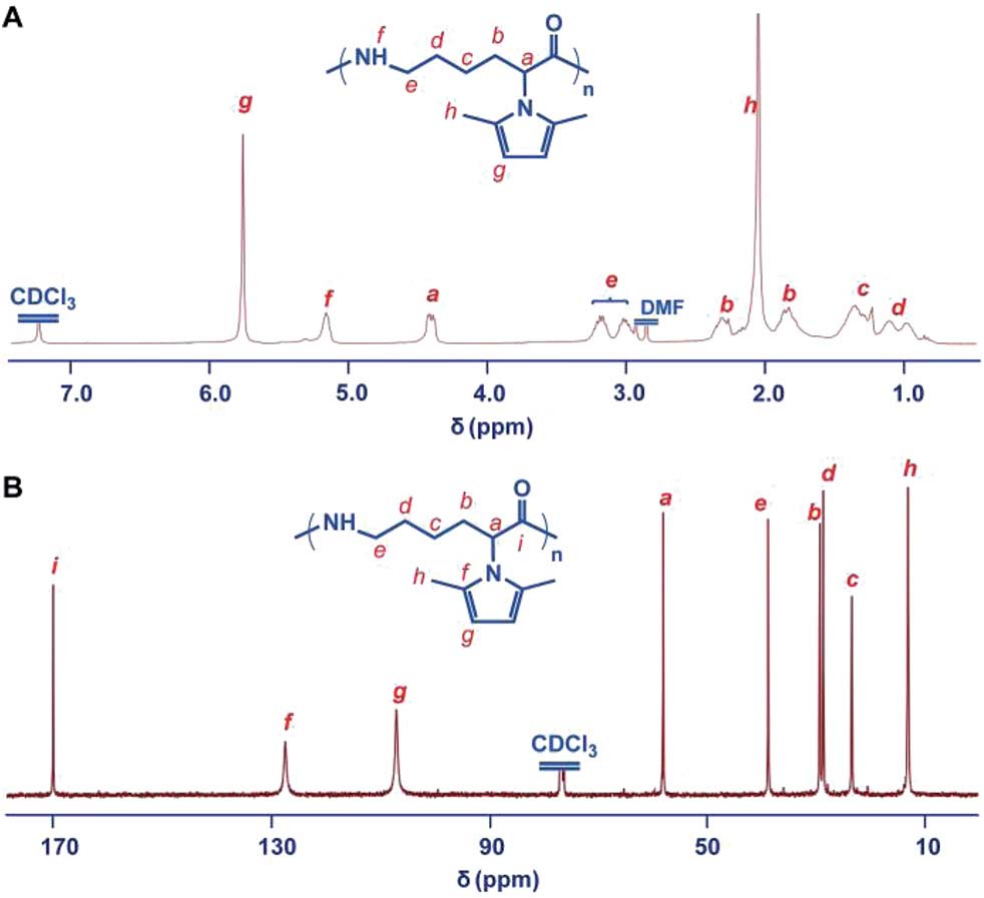
Structure characterization of the polymer of 2,5-dimethylpyrrole protected α-amino-ε-caprolactam monomer (PMPCL). (A) ^1^H NMR spectrum of PMPCL. (B) ^13^C NMR spectrum of PMPCL. The polymer was obtained with sodium (0.5%) in bulk at 260 °C. All spectra were measured in CDCl_3_ at room temperature.

The ^1^H NMR and ^13^C NMR spectra of the polymer are shown in Fig. 2. The ROP was further confirmed by the observation of the reduction of the C_α_–H resonance at 4.8 ppm of the monomer and the appearance of the corresponding broadened multiplets at 4.4 ppm of the polymer using ^1^H NMR spectroscopy. Both the ^1^H and ^13^C NMR spectra showed that the 2,5-dimethylpyrrole groups were stable towards the initiating and propagating species under these experimental conditions (Fig. 2). All of the obtained PMPCLs showed good solubility in common solvents, such as DMF, THF, chloroform and dichloromethane (Table S1†).

To show the advantages of the chemosynthetic method, random copolymerization was examined. The copolymerization of MPCL **3** with commercially available ε-caprolactam (CL) resulted in >70% polymer yield (Table S2†). ^1^H NMR analysis confirmed the presence of ε-caprolactam units (Fig. S8†). The CL/pyrrole-CL unit ratio in the copolymer could be calculated from the ^1^H NMR spectrum. As listed in Table S2,† the CL/MPCL unit ratio in the copolymer was close to the initial comonomer ratio.

### Removal of the 2,5-dimethylpyrrole groups

**Fig. 3:**
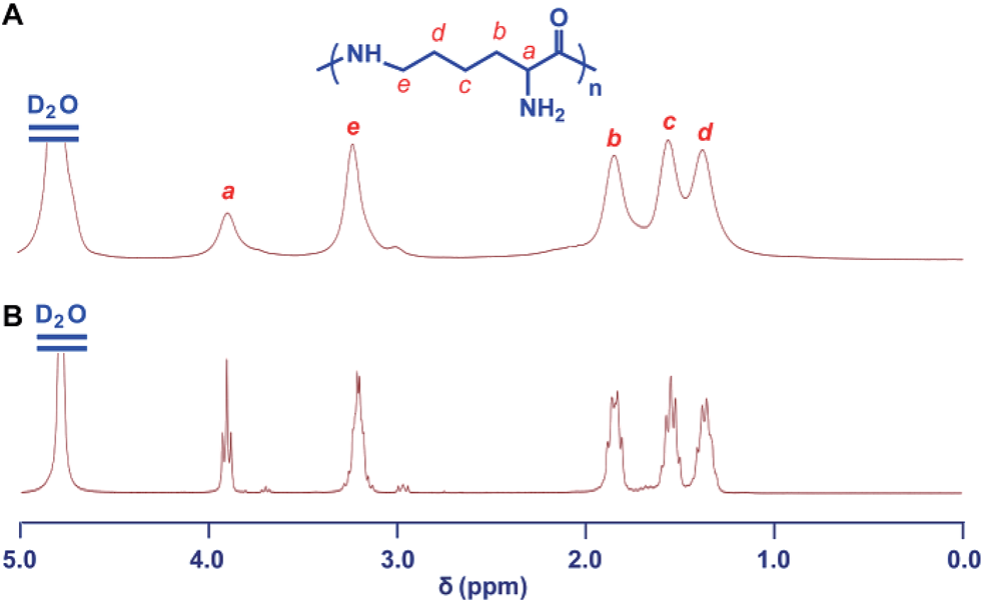
Structure characterization of ε-PL. ^1^H NMR spectra of chemosynthetic ε-PL (A) and biosynthetic ε-PL (B). All spectra were measured in D_2_O, pH 2 at room temperature.

**Fig. 4:**
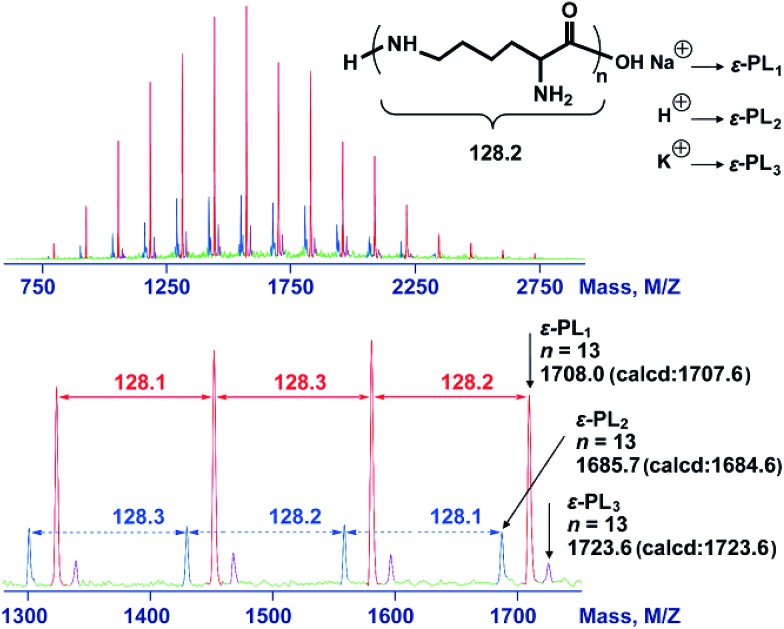
MALDI-TOF-MS spectra of the chemosynthetic ε-PL. The sample was obtained by ROP followed by the removal of the protecting group. The red peaks correspond to ε-PL_1_. The blue peaks correspond to ε-PL_2_. The purple peaks correspond to ε-PL_3_.

The 2,5-dimethylpyrrole protecting groups were removed by reaction with hydroxylamine/triethylamine in THF under a nitrogen atmosphere to yield the corresponding ε-PL. After deprotection, the signals in the ^1^H NMR and ^13^C NMR spectra (Fig. 3A and S9†) corresponding to the 2,5-dimethylpyrrole groups disappeared entirely, indicating the complete removal of the protecting groups. To illustrate the similarity between the polymers obtained from this new process and those obtained with traditional biosynthetic procedures, the ^1^H NMR spectrum of biosynthetic ε-PL was recorded (Fig. 3B). The ^1^H NMR spectrum again showed a very similar peak profile, indicating that ε-PL had been synthesized successfully *via* ROP. Fig. 4 shows a typical MALDI-TOF spectrum of the chemosynthetic ε-PL. The highest series of peaks were separated by the molecular weight of one lysine unit (128.2) and could be assigned to a series of ε-PLs with an NH_2_ unit at the initiating terminal and a –COOH unit at the capping terminal along with the lysine main-chain repeating sequence. Besides the highest series, there were two minor series of peaks, which are attributed to other ionized cations, such as H^+^ or K^+^. These results indicate that the ε-PL obtained through ROP possessed highly controlled terminal groups as well as a nearly perfect main-chain structure.

### Quantum chemical calculations

**Fig. 5:**
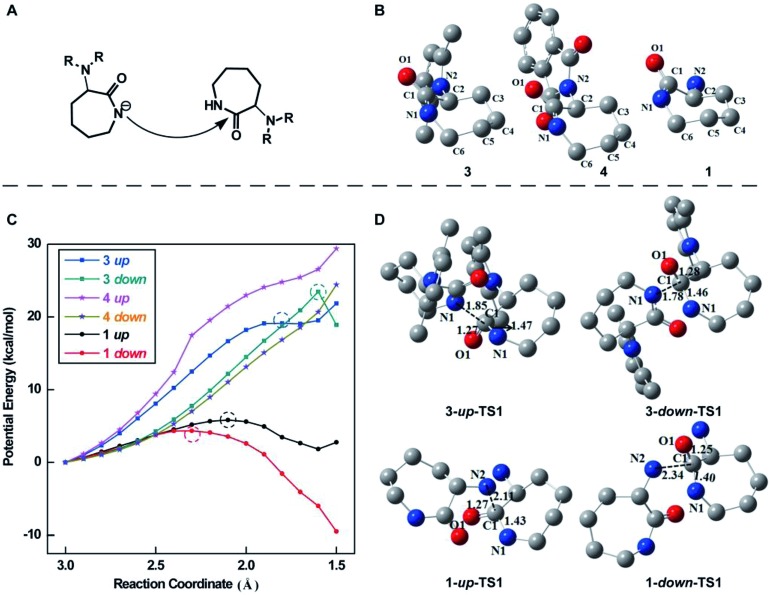
(A) Illustration of the initialization reaction of the lactam monomer during ROP. (B) The most favorable (*i.e.* the lowest energy) conformations of monomers **3**, **4** and **1**. (C) The potential energy profiles of the nucleophilic attack step along the reaction coordinate, which is defined as the distance between the nucleophilic N atom and the carbonyl C atom. The points with the highest energy for **3** and **1** are denoted using the dashed circle. (D) Optimized transition state structures of monomers **3** and **1** in the nucleophilic attack step. All hydrogen atoms are omitted for clarity.

It has already been demonstrated that the ROP of lactam monomers in the presence of sodium involves the generation of “activated monomers”, which react with monomeric lactam to form a primary amine-anion, which in turn regenerates deprotonated lactam c (Scheme S1†).^49^ To better understand the importance of protecting groups in the successful synthesis of the linear ε-PL, we performed quantum chemical calculations of the initialization reaction (Fig. 5A) during the ROP of monomers **3** and **4**. The most favorable equilibrium conformation of these monomers is the “chair” conformation (Fig. 5B), with the C2–C1(=O1)–N1–C6 segment being almost planar. We first calculated the potential energies along the nucleophilic attack reaction pathway, in which the nucleophilic N atom (N1 for **3** and **4**) gradually approached the carbonyl C atom (C1) from 3.0 to 1.5 Å. The potential energy profiles for **3** exhibited the highest energy point whatever the side of the nucleophilic attack (*i.e. up* or *down* of the plane), while the profiles for **4** increased monotonously (Fig. 5C). These results suggested that the nucleophilic attack reaction for **3** could take place but not for **4**. Next, we successfully delineated the free energy profiles of the whole initialization reaction for **3** including the nucleophilic attack and the ring-opening of the caprolactam ring. The optimized geometries of the transition state of the nucleophilic attack step for **3** are displayed in Fig. 5D. The nucleophilic N atom of **3** could attack the carbonyl C1 atom from “*up* or *down*” of the C2–C1(=O1)–N1–C6 plane. The calculations indicated that the *down* approach is thermodynamically more favored by over 5.5 kcal mol^–1^. Furthermore, we attempted to locate the transition state in the nucleophilic attack step for **4**, unfortunately without success. This also indicated that the initialization reaction of **3** could proceed, but does not for **4**. This observation is consistent with the experimental results. Similarly, we performed calculations of the initialization reaction of α-amino (N_2_) in monomer **1**. The calculations indicated that the amino N_2_ of **1** was a better nucleophilic regent due to the lower activation free energy barrier in the nucleophilic step (Fig. 5C and D), confirming that the polymerization of monomer **1** would indeed not result in the formation of linear polymers.

### Properties of ε-PL

To evaluate whether the chemosynthetic ε-PL possesses similar performances to the biosynthetic analogue, the main properties of chemosynthetic ε-PL, including its p*K*_a_ value, optical activity, secondary structure and cell cytotoxicity, were investigated and compared with those of the biosynthetic ε-PL. The p*K*_a_ of chemosynthetic ε-PL, determined by conventional potentiometric titration, was ∼7.5, which is close to that of biosynthetic ε-PL (p*K*_a_ = 7.6). Different from α-PL, which showed obvious cationic characteristics (p*K*_a_ = 9 to 10), the lower p*K*_a_ values of the chemosynthetic ε-PL will facilitate its application.^43^

The secondary structure of ε-PL was investigated using FTIR spectroscopy. There was an amine **I** absorption at 1670 cm^–1^ for the chemosynthetic ε-PL (Fig. S10†), indicating that the chemosynthetic ε-PL adapted a β-turn conformation in the solid state.^48^

**Fig. 6:**
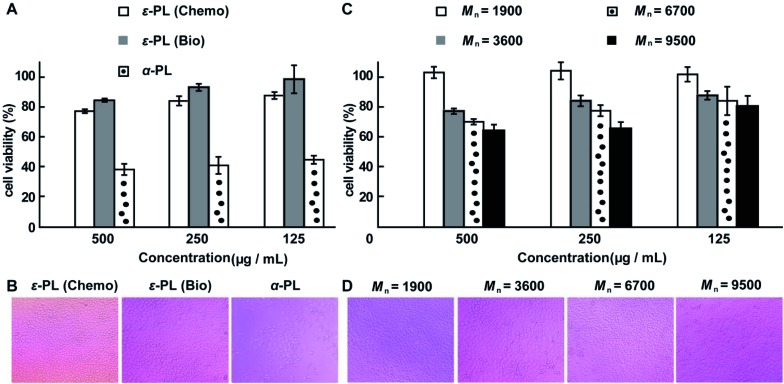
MTT cytotoxicity assay. (A) Viability of L929 cells, as determined by the MTT assay following 48 hours treatment with ε-PL and α-PL, separately. (B) Microscopy images of L929 cells treated with 0.5 mg mL^–1^ of polymer. (C) MTT was used to detect the toxicity of the different ε-PLs as a function of molecular weight at various concentrations. (D) Microscopy images of L929 cells treated with 0.25 mg mL^–1^ of ε-PL. The MTT cytotoxicity assay showed no toxicity to L929 cells at concentrations of 0.125 to 0.25 mg mL^–1^ of ε-PL with varying molecular weights. The molecular weight values of ε-PL are estimated from the values of their corresponding acetylated versions after subtraction of the acetyl groups.

The cytotoxicity of the chemosynthetic ε-PL was evaluated against L929 cells by the MTT assay. α-PL and biosynthetic ε-PL were used as controls. The chemosynthetic ε-PL exhibited a similar cytotoxicity to the biosynthetic analogue but had a much lower cytotoxicity compared to that of α-PL at concentrations of 0.125 to 0.5 mg mL^–1^ (Fig. 6A). The optical images show that most of the cells died when incubated in a solution of 0.5 mg mL^–1^ α-PL (Fig. 6B), but the L929 cells featured a good growing status for the chemosynthetic ε-PL system, which was quite similar to that of the biosynthetic ε-PL, indicating the high compatibility of the chemosynthetic ε-PL.

To expand the application of ε-PL in the field of biomaterials, ε-PL with varying molecular weights is needed. Therefore, to further illustrate the advantages of this new chemosynthetic procedure and to understand whether the increase in the molecular weight of ε-PL changed its biocompatibility, chemosynthetic ε-PL samples featuring different molecular weights were selected for the MTT cytotoxicity assay. Chemosynthetic ε-PL with *M*_n_ 9500 showed no obvious cytotoxicity at concentrations up to 250 μg mL^–1^ after 48 hours incubation (Fig. 6C and D). The excellent cell compatibility along with the diverse molecular weights render chemosynthetic ε-PL a useful material for the design of gene and drug delivery systems.

The measurement regarding the optical activity showed [*α*]25 °C578 nmHg = +23° for biosynthetic ε-PL, while the [*α*] value of the chemosynthetic analogue was found to be 0°. This result indicated that racemization occurred during the polymerization in the presence of catalytic amounts of sodium. A similar phenomenon was also observed in other sodium-catalyzed polymerizations.^47^ Basically, the secondary conformations of the polypeptide in solution are strongly dependent on the chirality of the polypeptide.^4^,^31^ Although the above-mentioned results showed that the chemosynthetic ε-PL exhibited a similar p*K*_a_ value and low cytotoxicity as the biosynthetic analogue, we are pretty sure that the stereochemistry change would influence the secondary structure as well as the condensed structure of ε-PL in solution or in the solid state.

## Conclusions

In conclusion, we have demonstrated a facile synthesis method for linear ε-PL with varying molecular weights based on ROP. Experimental and theoretical studies reveal that the appropriate selection of a protecting group proves to be crucial for the efficient synthesis of ε-PL. Unprotected α-amino-ε-caprolactam or monomers with common protecting groups, such as Boc and Cbz, were not effectively polymerized to afford linear ε-PL. The use of protecting groups featuring large steric hindrance, such as dibenzyl or phthalimide, did not lead to ε-PL either. Unlike ε-PL from the fermentation process, the chemosynthesis of ε-PL *via* ROP can produce polymers with diverse molecular weights and chemical compositions. This feature proves to be extremely valuable for potential future applications. Moreover, different from α-PL *via* the NCA route, the strategy presented here is phosgene-free, critical for a safe and feasible large-scale production process. These chemosynthetic ε-PLs offer highly promising properties for a variety of applications, *e.g.* as a cosmetic additive or in biodegradable products. Furthermore, this methodology may provide access to the production of γ-polyglutamate and β-poly(aspartic acid) from glutamic acid and aspartic acid monomers, respectively.

## Supplementary Material

Supplementary information
