# Supplementary material for: New chemosynthetic route to linear ε-poly-lysine
Source: Chem Sci. 2015 Jul 28;6(11):6385–91. doi: 10.1039/c5sc02479j (PMC6054053; doi:10.1039/c5sc02479j)
Supplement: Supplementary file 1 [file SC-006-C5SC02479J-s001.pdf]

Electronic Supplementary Material (ESI) for Chemical Science.
This journal is © The Royal Society of Chemistry 2015

Supporting information

New Chemosynthetic Route to Linear ε-Poly-Lysine

Youhua Tao*, Xiaoyu Chen, Fan Jia, Shixue Wang, Chunsheng Xiao, Fengchao Cui,

Yunqi Li, Zheng Bian, Xuesi Chen, Xianhong Wang*

Key Laboratory of Polymer Ecomaterials, Changchun Institute of Applied Chemistry, Chinese Academy of Sciences, Renmin Street 5625, Changchun 130022, People's Republic of China

* Corresponding author. E-mail: youhua.tao@ciac.ac.cn, xhwang@ciac.ac.cn;

Web: http://youhuatao.weebly.com/

Materials and methods.. S2

Synthesis of Monomers.. S3

Polymerization.. S8

Deprotection.. S8

Acetylation of ε-PL with Acetic Anhydride.. S9

Computational methods.. S9

Cell viability assays.. S10

Supplementary Scheme.. S11

Supplementary Tables.. S12

Supplementary Figures.. S14

References.. S25

Materials and Methods

All reagents and solvents obtained from commercial sources were used as received without further purification unless stated otherwise. L-Lysine monohydrochloride (99%) was obtained from Chengdu Baishixing Chemical Industrial Co., Ltd. Biosynthetic *ε*-PL with an approximate molecular weight of M_n ~ 3000 g/mol was received from Silver-Elephant Bio-engineering Co., Ltd, China. *α*-PL (molecular weights 1000–5000 g/mol) was purchased from Sigma-Aldrich. The Spectra/Pore® dialysis membranes (MWCO 1000) were purchased from Spectrum Laboratories Inc., USA.

^1H NMR spectra and ^{13}C NMR spectra were recorded on a Bruker AV-300 spectrometer and a Bruker AV-400 spectrometer, respectively. Matrix-assisted laser desorption/ionization time-of-flight mass spectroscopy (MALDI-TOF/MS) was performed on a Bruker atuoflex III mass spectrometer in linear, positive ion mode equipped with 355nm smartbeam laser. The matrix was dithranol (DIT), and solvent was H_2O. Gel permeation chromatography (GPC) was conducted on a system composed of a Waters 2414 Refractive Index Detector equipped with a series of linear Shodex columns (KD-802.5, KD-804 and KD-G). The system was operated with DMF (containing 0.02M LiBr) as the eluent at a flow rate of 1 mL/min at 50 ^{o}C and calibrated with polymethyl methacrylate standards in the molecular weight range from 1860 to 3.3×10^5 Da. The pK_a of *ε*-PL was determined by potentiometric titration as described earlier.[1] Analyses of the optical activity were performed with a Perkin-Elmer 241 polarimeter with a mercury vapor lamp (578 nm) at 25 °C. ESI- MS was

conducted on a system composed of a Waters Quattro Premier XE. The system was operated with Full Scan function type, 3.0kV Capillary, 20V Cone, 110℃ Source Temperature, 80L/Hr Cone Gas Flow, 380 °C desolvation temperature and 600 L/Hr Desolvation Gas Flow. Fourier transform infrared spectra were performed on a Bruker TENSOR-27 spectrophotometer.

Synthesis of Monomers

Synthesis of lysine methyl ester dihydrochloride

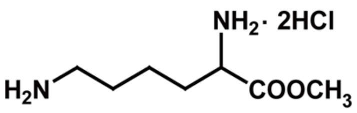

lysine methyl ester dihydrochloride was synthesized by a modified procedure as described earlier.[2] Briefly, (*S*)-lysine monohydrochloride (36.5 g, 0.2 mol) was dissolved in methanol (500 mL) and cooled in an ice bath, then thionyl chloride (42 mL, 0.6mol) was added dropwise over 30 min. The reaction mixture soon became homogeneous and was stirred for 20 min at 0 °C. After heating at reflux for 4 h, the solution was concentrated *in vacuo* and the crude product was precipitated out as white crystal. The crude product was collected by filtration and washed with ethyl ether, and then dried under vacuum to afford the product (47.25 g, 92.5%). ^{1}H NMR (300 MHz, D_2O) δ 4.08-4.03 (t, 1H), 3.743 (s, 3H), 2.92-2.87 (t, 2H), 1.90-1.87 (m, 2H), 1.60-1.55 (m, 2H), 1.43-1.34 (m, 2H).

Synthesis of *α*-amino-*ε*-caprolactam (1)

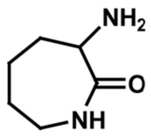

lysine methyl ester dihydrochloride (23.3g, 0.1mol) was dissolved in 400 mL methanol together with NaOH (12g, 0.3mmol). The mixture was stirred at room temperature for 24 hours. The crude product was purified by recrystallization from ethylacetate to get *α*-amino-*ε*-caprolactam as a white powder (7.39 g, 57.8%). ^{1}H NMR (300 MHz, DMSO) δ 3.44 (d, 1H); 3.06 (m, 2H); 1.83 (m, 1H); 1.64 (m, 2H,); 1.56(m, 1H); 1.36-1.13 (m, 2H). ^{13}C NMR (D_2O, 100 MHz) *δ*: 181.1, 52.8, 41.4, 32.6, 27.9, 27.7. ESI-MS: calculated for $C_6H_{12}N_2O$, 127.9; found for M^+Na ($C_6H_{12}N_2O$ Na), 150.9.

Synthesis of *tert*-butoxycarbonyl (Boc) protected *α*-amino-*ε*-caprolactam monomer (2a)

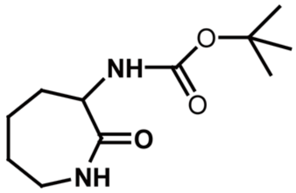

To the solution of *α*-amino-*ε*-caprolactam (0.64g, 5mmol) and K_2CO_3 (0.69g, 5mmol) in 5mL of tetrahyrofuran (THF) cooled in an ice bath, di-*tert*-butyl dicarbonate (1ml, 7.7mmol) was added dropwise. After 20 min, the ice bath was removed and the reaction was allowed to proceed at room temperature for 18 hours. The precipitate was removed by filtration, then THF was evaporated, and the crude product was

purified by column chromatography using petroleum ether and ethyl acetate (9:1) as eluent to afford the product as white solid (0.81 g, 71.1%). ^{1}H NMR (300 MHz, $CDCl_3$) δ 6.45 (s, 1H); 5.93 (s, 1H); 4.27 (m, 1H); 3.25 (s, 2H,); 2.05(m, 2H); 1.81(m, 2H); 1.51(m, 2H); 1.45(s, 9H). ^{13}C NMR ($CDCl_3$, 100 MHz) *δ*: 175.87, 154.90, 78.97, 52,91, 41.67, 31.92, 28.61, 27.84.

Synthesis of carbobenzyloxy (Cbz) protected *α*-amino-*ε*-caprolactam monomer (2b)

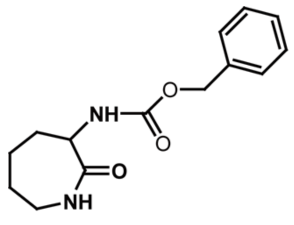

To the solution of *α*-amino-*ε*-caprolactam (1g, 7.6mmol) and $NaHCO_3$ (0.95g, 11.4mmol) in a mixed solvent of THF/water(10 mL/4 mL) cooled in an ice bath, benzyl chloroformate (1mL, 7.7mmol) was added dropwise. After 20 min, the ice bath was removed and the reaction was allowed to proceed at room temperature for 18 hours. After evaporation of solvent under vacuum, the crude product was purified by recrystallization from diethyl ether and chloroform to get white solid (1.75 g, 85.4%). ^{1}H NMR (300 MHz, $CDCl_3$) δ 7.37 (m, 5H); 6.17 (m, 2H); 5.12 (s, 2H); 4.38 (m, 1H,); 3.25(m, 1H); 2.11 (dd, 2H); 1.86(m, 2H); 1.57-1.39(m, 2H). ^{13}C NMR ($CDCl_3$, 100 MHz) *δ*: 175.52, 155.27, 136.35, 128.18, 127.70, 127.68, 66.26, 53.32, 41.61, 31.65, 28.49, 27.73.

Synthesis of 2,5-dimethylpyrrole protected α-amino-ε-caprolactam (MPCL) monomer (3)

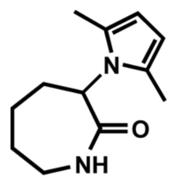

Acetic acid (0.22 mL, 3.9 mmol) and 2,5-hexanedione (0.46 mL, 3.9 mmol) were slowly added to a solution of *α*-amino-*ε*-caprolactam (0.5 g, 3.9 mmol) in methanol (25 mL). The mixture was stirred at 50 °C under nitrogen for 12 hours, and subsequently concentrated *in vacuo*. 40 mL of a NaOH solution (4M) was added and the product was extracted with DCM (3 x 50 mL). After evaporation of solvent *in vacuo*, the crude product was subjected to column chromatography using petroleum ether and ethyl acetate (3:1) as eluent to afford the product as a white solid (0.54 g, 74.6%). ^{1}H NMR (300 MHz, $CDCl_3$) δ 7.46 (s, 1H); 5.85 (s, 2H); 4.91 (d, 1H); 3.29 (s, 2H,); 2.32(s, 6H); 2.19-1.92 (m, 4H); 1.73-1.54(dd, 2H). ^{13}C NMR ($CDCl_3$, 100 MHz) *δ*: 174.6, 128.3, 106.2, 77.6, 58.0, 42.3, 32.3, 29.7, 29.0,14.4. ESI-MS: calculated for $C_6H_{12}N_2O$, 206.1; found for M^+Na ($C_{12}H_{18}N_2O$ Na), 229.1.

Synthesis of phthalimide protected *α*-amino-*ε*-caprolactam monomer (4)

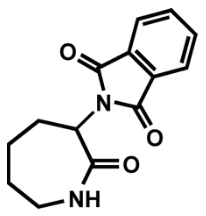

α-amino-*ε*-caprolactam (0.5g, 4mmol) and Phthalic anhydride (0.69g, 4.7mmol) was dissolved in dioxane (20mL). The mixture was stirred at 120 °C under nitrogen. After 2h, the mixture was concentrated under vacuum and the reaction was allowed to proceed in DMF at 150 °C for 2 hours. Then 40 mL water was added to the mixture and the mixture was extracted with ethyl acetate (100 mL×3). The organic layer was dried over anhydrous Na_2SO_4.The solvent was evaporated under vacuum and the residue was purified by column chromatography using petroleum ether/ ethyl acetate (v:v = 1:1) as eluent to afford the product as white solid (0.69 g, 68.1%). ^1H NMR (300 MHz, $CDCl_3$) δ 7.81-7.28 (m, 4H); 6.88 (s, 1H); 4.89(d, 1H); 3.24 (s, 2H,); 2.63-2.15 (dd, 2H); 2.09-1.98(dd, 2H); 1.70(m, 2H); 1.47(m, 2H).

Synthesis of Dibenzyl protected α-amino-ε-caprolactam monomer (5)

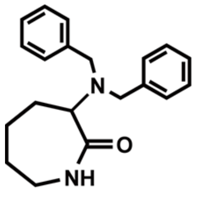

α-amino-ε-caprolactam (1.28g, 10mmol), benzyl chloride (2.3ml, 20mmol) and K_2CO_3 (2.0g, 15mmol) was dissolved in acetonitrile (40mL). The mixture was stirred at 80 °C under nitrogen. After 2h, the mixture was concentrated under vacuum and the residue was purified by column chromatography using petroleum ether/ ethyl acetate (v:v = 1:2) as eluent to afford the product as white solid (2.52 g, 81.1%). ^1H NMR

(300 MHz, $CDCl_3$) δ 7.41-7.32 (m, 10H); 5.83 (s, 1H); 4.07-3.92(dd, 4H); 3.45 (d, 1H,); 3.09-2.90 (m, 2H); 1.99(d, 2H); 1.70(m, 2H); 1.45(s, 2H).

Polymerization.

In a typical experiment, monomer or combined monomers (1g) and sodium (5 mg) were weighted and added to a flame-dried polymerization tube. The mixture was purged with nitrogen for 30 min and the polymerization tube was subsequently placed into an oil bath with the temperature adjusted at 145 °C. The reaction mixture was stirred for 15 min at this temperature to allow *in situ* formation of a sodium-lactam salt. The temperature was then raised and maintained at 260 °C and the polymerization was allowed to proceed for a total of 2 hours under nitrogen. After cooling down to room temperature, the polymer was ground to a powder, dissolved in DMF and precipitated into water.

Deprotection.

A sample of 0.75g of PMPCL was dissolved in 150 mL of THF. Hydroxylamine hydrochloride (5.02 g, 20 eqv.), triethylamine (5.2 mL) and water (60 mL) were added to the above mixture under nitrogen. The mixture was then heated at 80 °C for 24 hours. Afterwards, another portion of hydroxylamine hydrochloride (1.67 g) and triethylamine (1.7 mL) were added. The solution was further heated at 80 °C until TLC monitoring confirmed complete conversion of the starting material. The reaction

mixture was then allowed to cool to room temperature and quenched by pouring it into a solution of ice-cold NaOH (6M). The mixture was concentrated and dialyzed (MWCO 1000, Spectrum Laboratories, Inc.) against water for a total of 3 days to yield the final polymer (35%~62% yield). ^1H NMR (300 MHz, D_2O) δ: 3.74(s, 1H); 3.07(s, 2H); 1.69(s, 2H); 1.41(s, 2H); 1.22(s, 2H). ^{13}C NMR (D_2O, 100 MHz) *δ*: 169.4, 53.3, 39.1, 30.5, 27.9, 21.7.

Acetylation of *ε*-PL with Acetic Anhydride

Acetylated *ε*-PL was synthesized by a modified procedure as described earlier.[3-4] Briefly, 1.84 g *ε*-PL was dissolved in 80 mL CH_3COONa solution (1 M), and the pH value was adjusted to 11 with the concentrated NaOH solution. The mixture was cooled with ice and acetic anhydride (in total: 6.1 g) was added in four equivalent portions. After each addition the pH value was adjusted to 11 with NaOH solution. The reaction mixture was stirred for 3 h between two addition steps. At the end of the reaction 30 mL of water were added and the solution was dialyzed against water. Lyophilization of the final solution yielded in a colorless solid product (1.2g). The ^1H-NMR spectrum of acetylated *ε*-PL in D_2O was shown in **Figure S13**.

Computational methods.

Calculations were performed using the B3LYP[5-10] density functional theory (DFT) with Gaussian09 suite of programs.[11] In order to confirm whether the transition state

exists or not, the potential energies along the nucleophilic attack reaction path were scanned at the B3LYP/6-31+G(d,p) level of theory. Frequency calculations were done to characterize the nature of the stationary points and to obtain the thermal corrections to Gibbs free energy at 298 K. Free energies of all stationary species at 298K discussed present will be relative to that of the infinitely separated monomers with and without proton in the nucleophilic N atom.

Cell viability assays.

L929 cells were seeded in a 96-well plate at 1×10^4 cells per well and incubated for 24 h at 37 °C. *ε*-PL were diluted with fresh medium (Dulbecco's Modified Eagle Medium supplemented with 10% (v/v) fetal bovine serum) to the desired concentrations and added to the corresponding well, respectively. After incubation for 48 h at 37 °C, cell viability was assessed using the standard MTT assay.

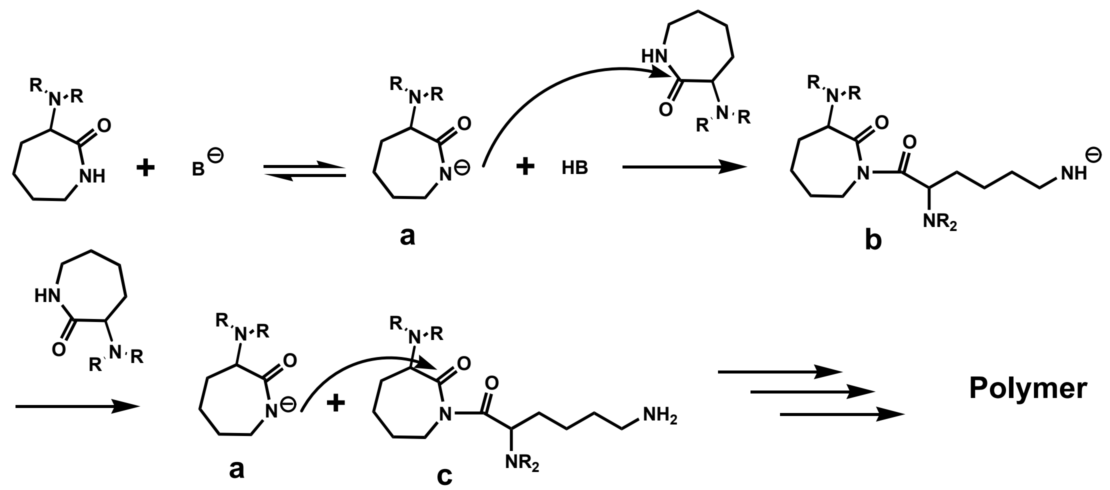

Scheme S1. Possible mechanism for the anionic ROP of lactam monomer.

Supplementary Tables

Table S1. Solubility of PMPCL and *ε*-PL [a]

sample	THF	DCM	$CHCl_3$	DMSO	DMF	Acetone	MeOH	EtOAc	H_2O	Ether
MPCL	S	S	S	S	S	S	S	S	I	I
PMPCL	S	S	S	S	S	S	I	I	I	I
ε-Poly-Lysine	I	I	I	S	S	I	S	I	S	I

[a] S= soluble; I= insoluble

Table S2. Bulk Ring-opening Copolymerization of MPCL with ε-caprolactam (CL) catalyzed by Sodium

Entry	CL/ MPCL Feed Ratio (mol%)	Yield (%)[c]	CL/ MPCL in the copolymer (mol%)[d]	M_n (kDa)[e]	M_w/M_n[e]
1[a]	40/60	88	32/68	4.8	1.1
2[a]	20/80	82	22/78	4.7	1.1
3[a]	10/90	76	13/87	5.6	1.2
4[b]	40/60	83	30/70	18.8	1.8
5[b]	20/80	78	26/74	15.4	1.7
6[b]	10/90	72	21/79	18.8	1.5

The reaction was performed in bulk in a 10 mL flame-dried polymerization tube.

[a]5% catalyst (Na) loading. [b]0.5% catalyst (Na) loading. [c]Isolated yield. [d]Determined by ^{1}H NMR.

[e]The number-average molecular weight (M_n) and distribution (M_w/M_n) were determined by GPC.

Supplementary Figures

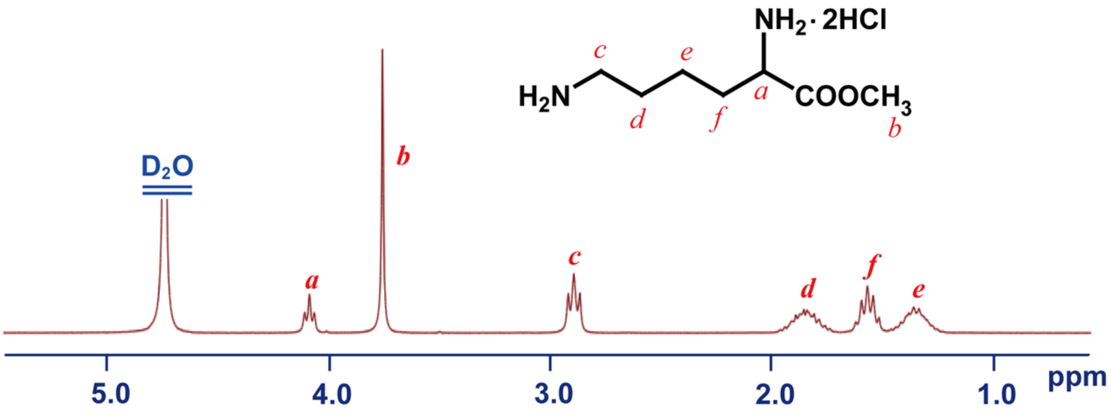

Figure S1. ^{1}H NMR spectrum of lysine methyl ester dihydrochloride (300 MHz, D_2O).

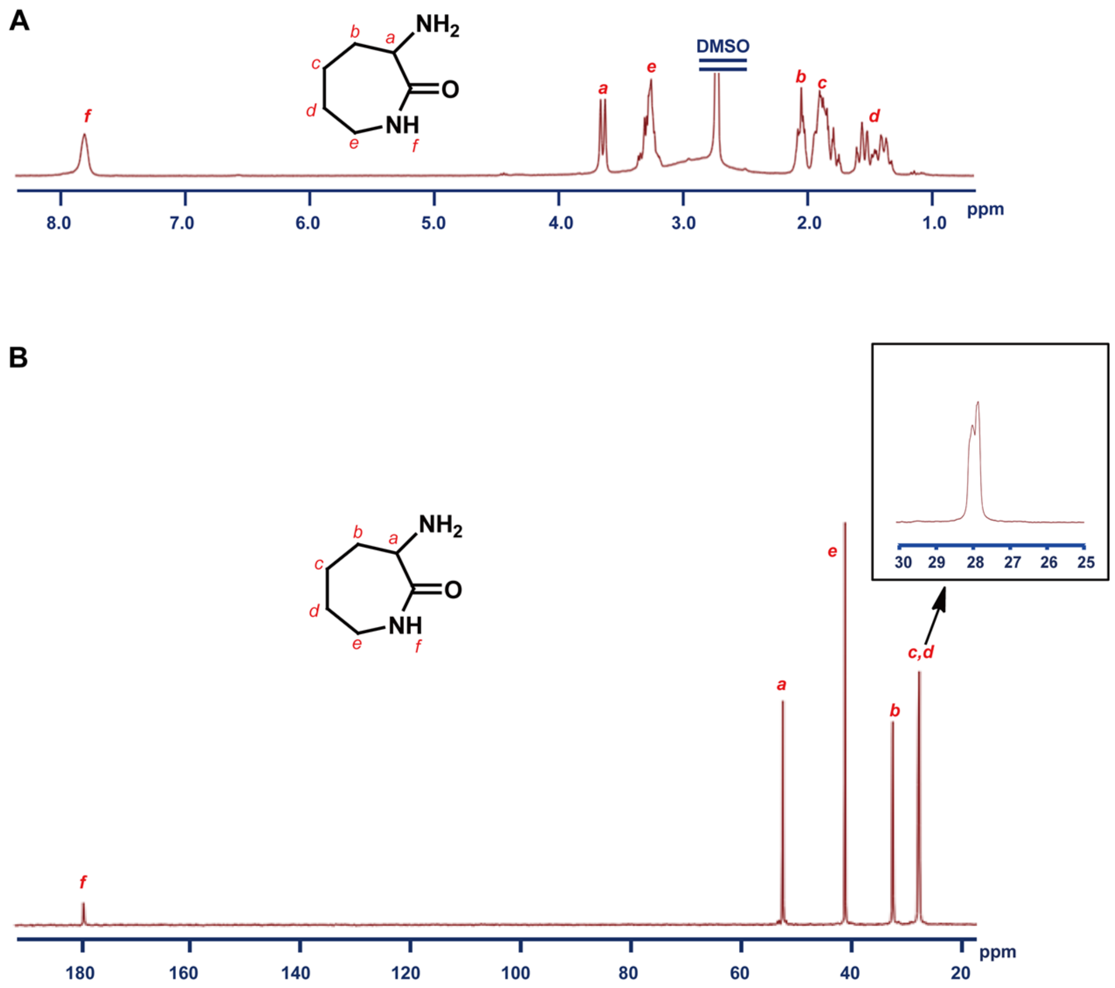

Figure S2. (**A**) ^{1}H NMR spectrum of *α*-amino-*ε*-caprolactam (300 MHz, DMSO). (**B**) ^{13}C NMR spectrum of *α*-amino-*ε*-caprolactam (100 MHz, D_2O).

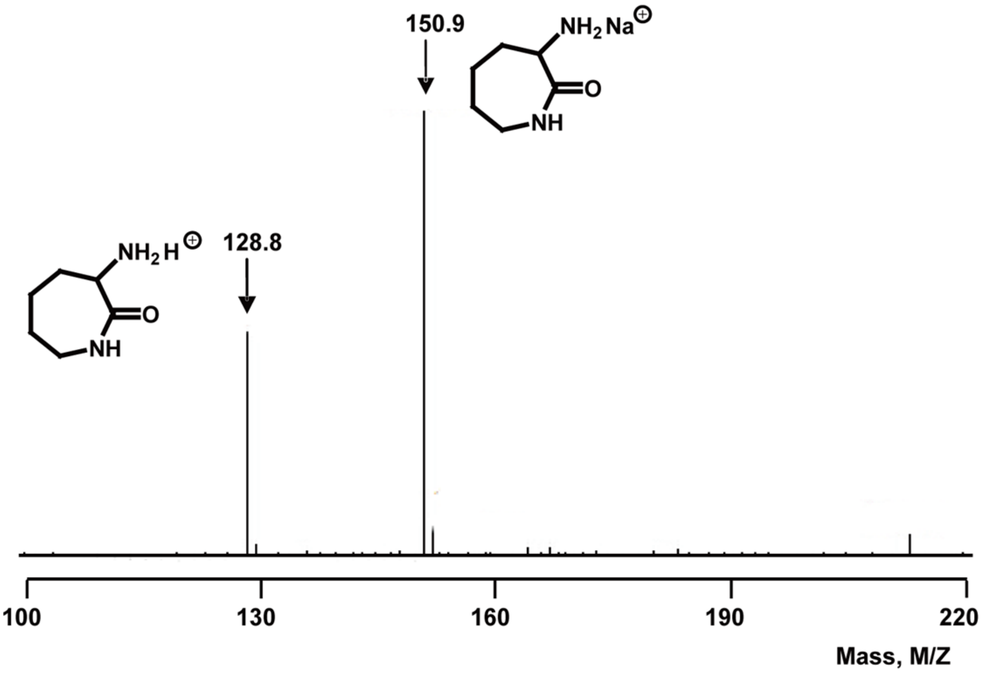

Figure S3. ESI-MS spectra of the *α*-amino-*ε*-caprolactam.

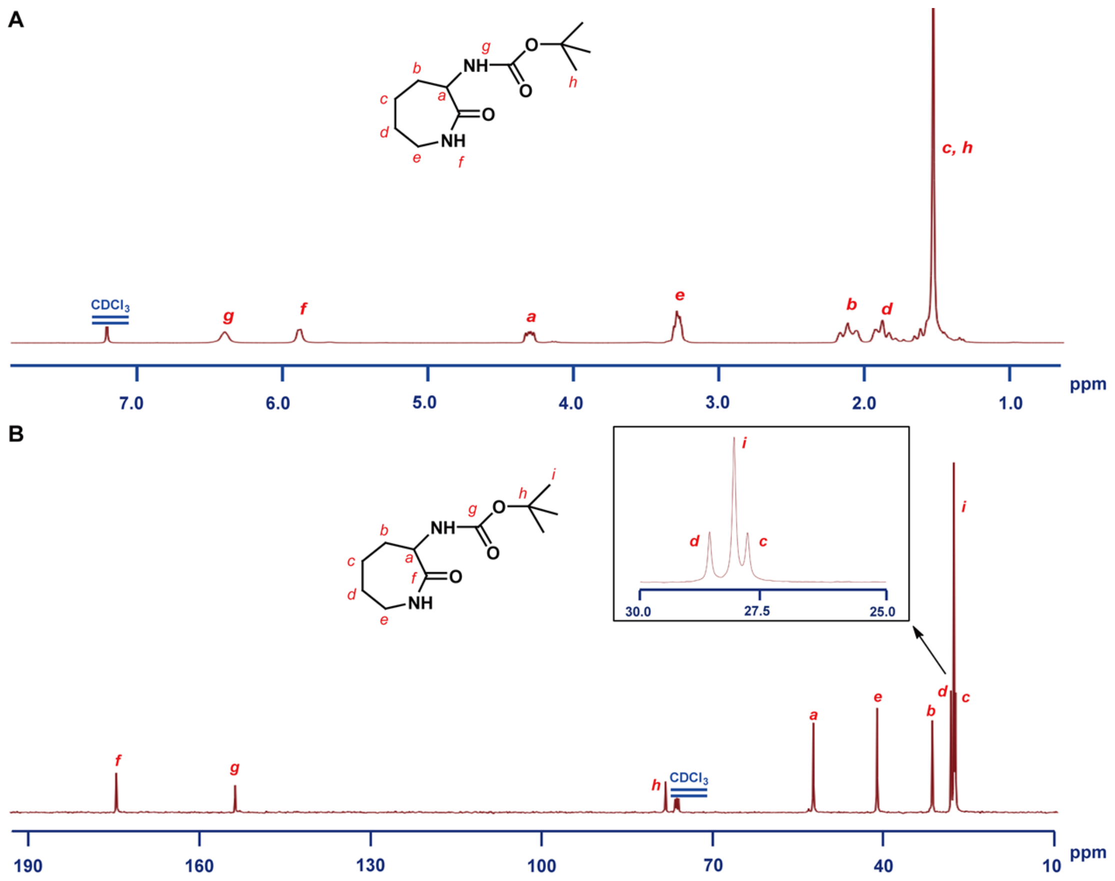

Figure S4. (**A**)^{1}H NMR spectrum of Boc protected *α*-amino-*ε*-caprolactam monomer **2a** (300 MHz, $CDCl_3$). (**B**) ^{13}C NMR spectrum of Boc protected *α*-amino-*ε*-caprolactam monomer **2a** (100 MHz, $CDCl_3$).

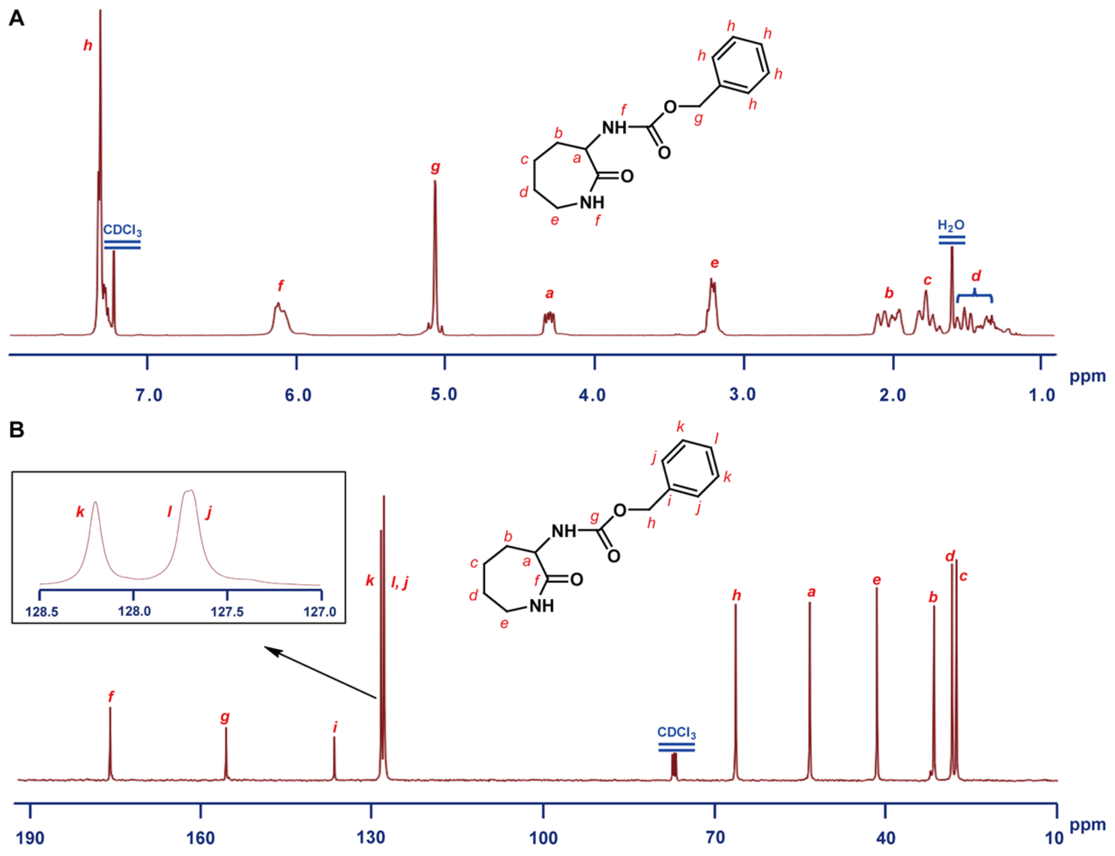

Figure S5. (**A**) ^{1}H NMR spectrum of Cbz protected *α*-amino-*ε*-caprolactam monomer **2b** (300 MHz, $CDCl_3$). (**B**) ^{13}C NMR spectrum of Cbz protected *α*-amino-*ε*-caprolactam monomer **2b** (100 MHz, $CDCl_3$).

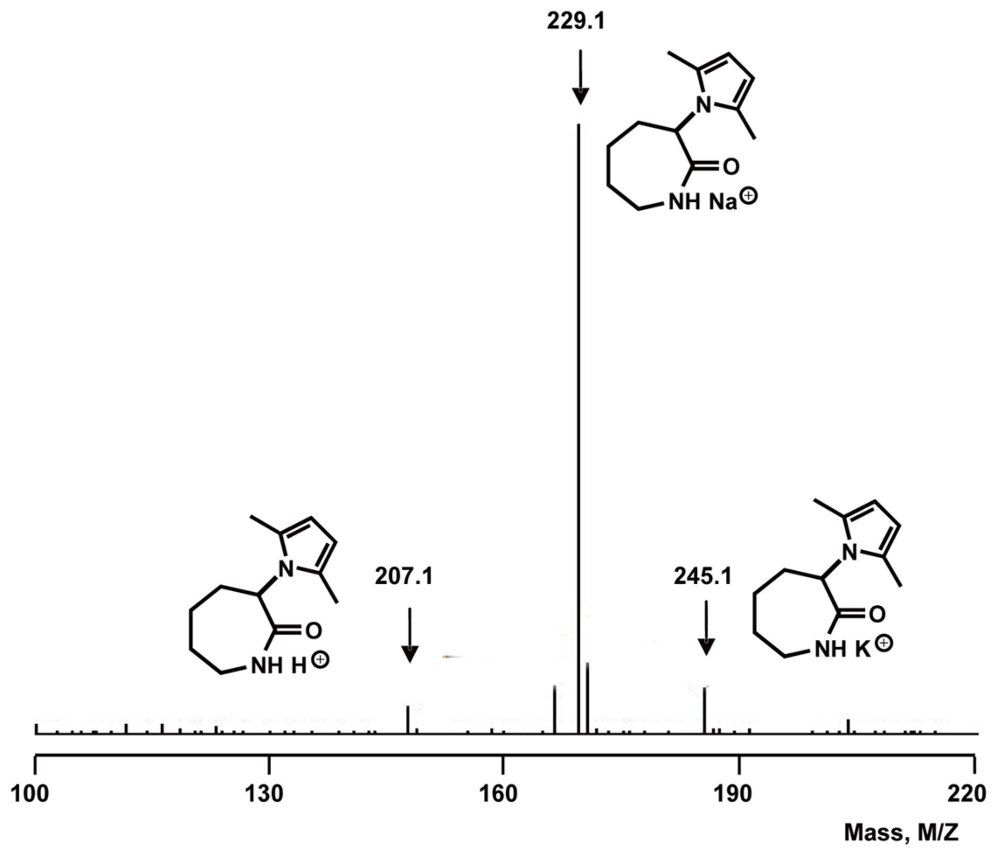

Figure S6. ESI-MS spectra of the 2,5-dimethylpyrrole protected *α*-amino-*ε*-caprolactam monomer.

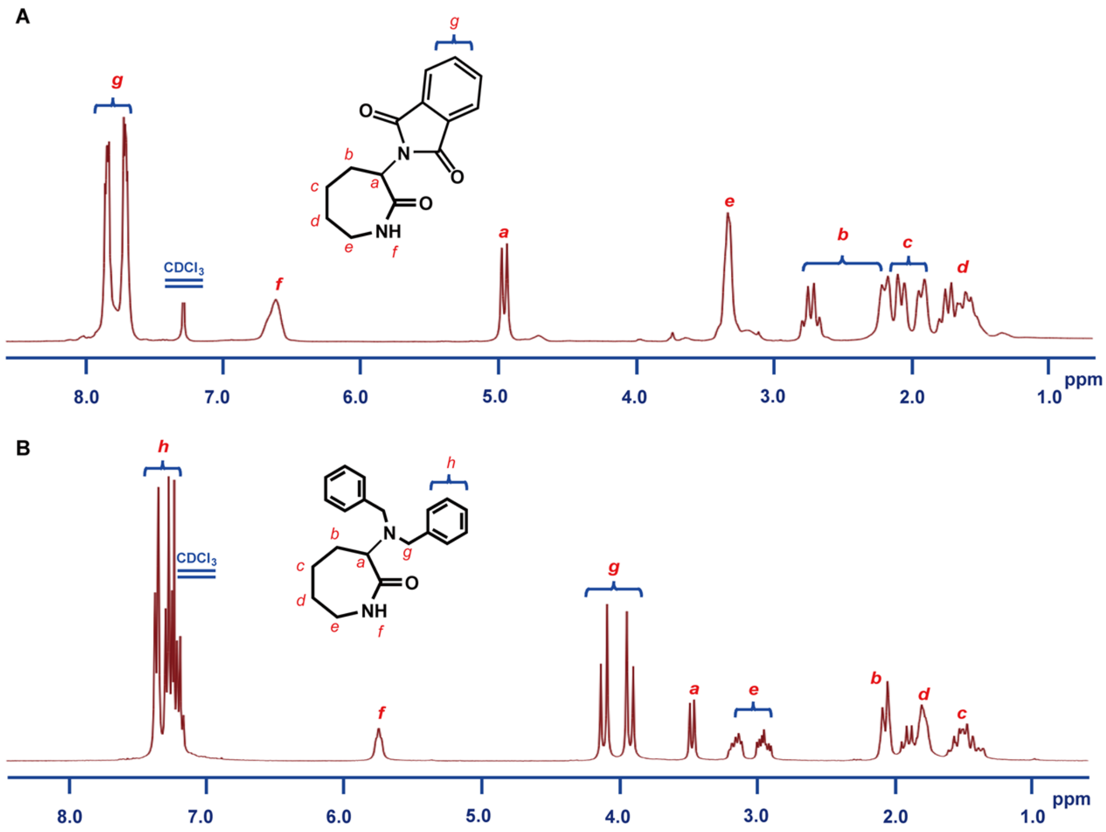

Figure S7. (**A**) ^{1}H NMR spectrum of phthalimide protected *α*-amino-ε-caprolactam monomer **4** (300 MHz, $CDCl_3$). (**B**) ^{1}H NMR spectrum of dibenzyl protected *α*-amino-*ε*-caprolactam monomer **5** (300 MHz, $CDCl_3$).

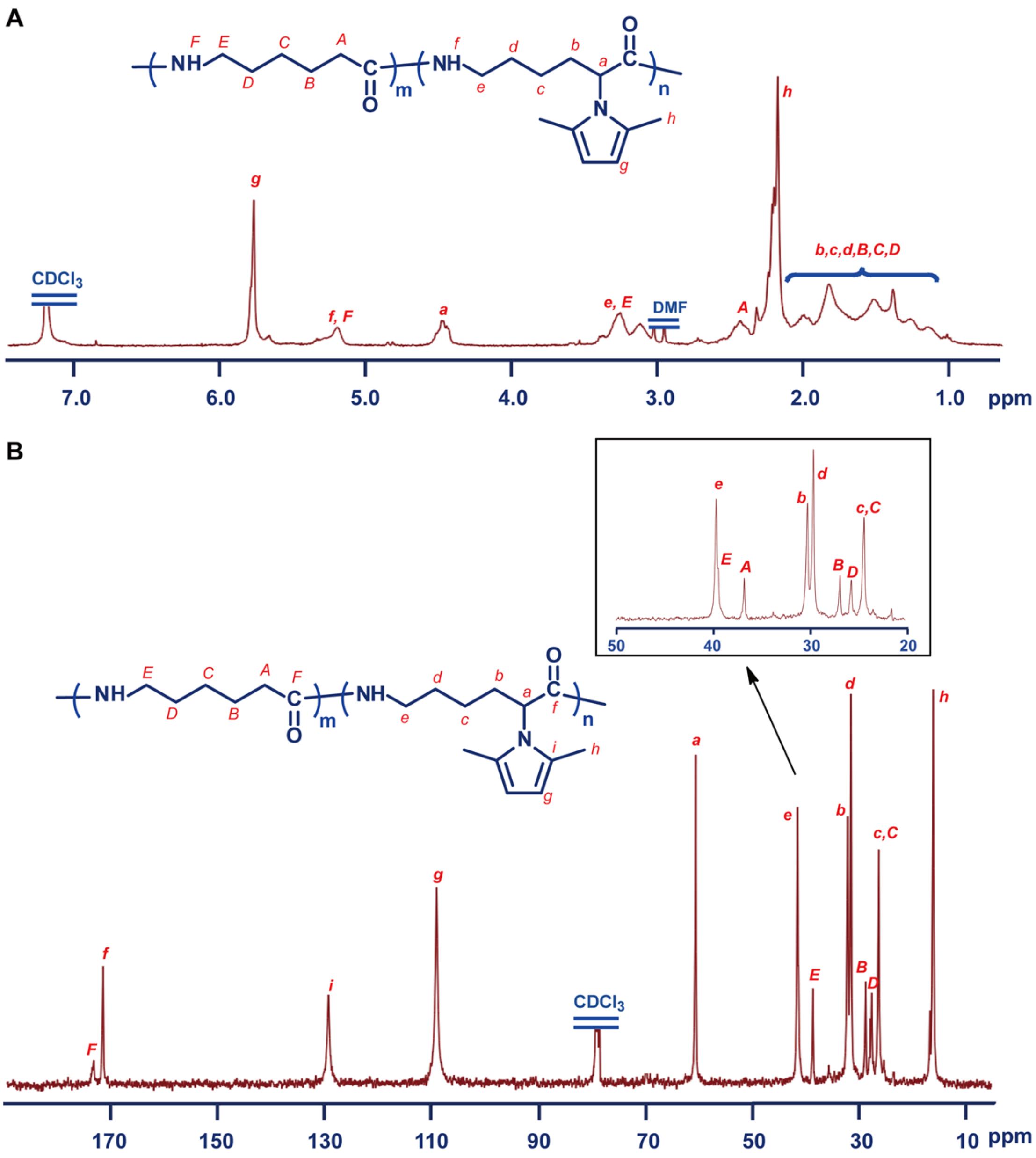

Figure S8. (A) ^{1}H NMR spectrum of copolymer of MPCL and caprolactam monomer (300 MHz, $CDCl_3$). (B) ^{13}C NMR spectrum of copolymer of MPCL and caprolactam monomer (100 MHz, $CDCl_3$).

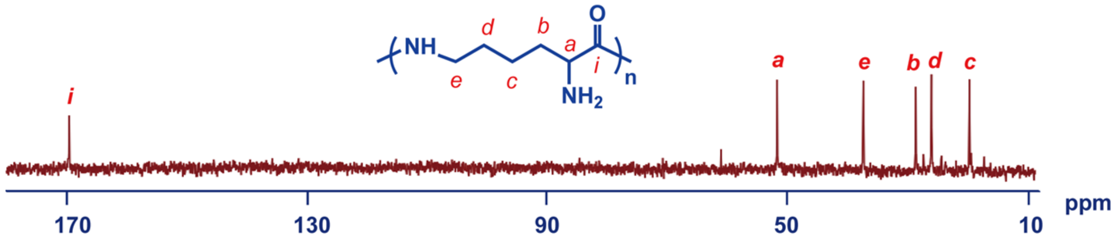

Figure S9. **Structure characterization of *ε*-PL.** ^{13}C NMR spectrum of chemosynthetic *ε*-PL. The polymer was purified by dialysis and lyophilized. All spectra were measured in D_2O, pH 2 at room temperature.

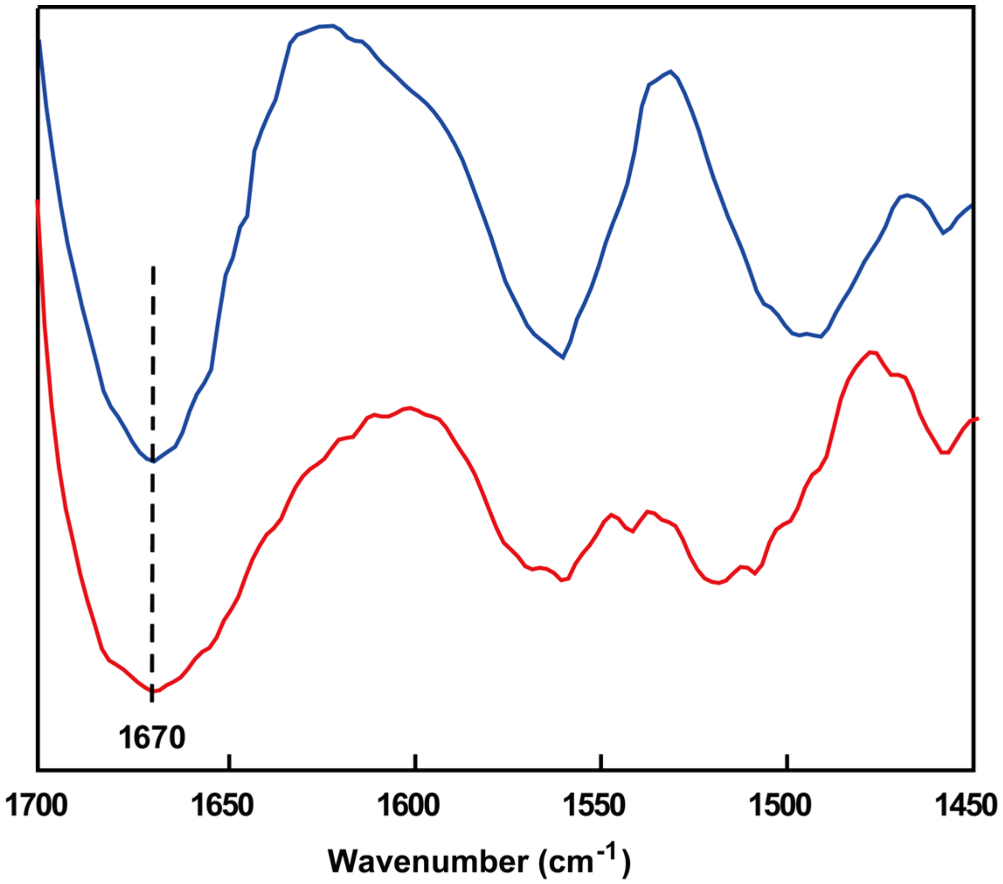

Figure S10. FTIR spectra of biosynthetic *ε*-PL (blue line) and chemosynthetic *ε*-PL (red line).

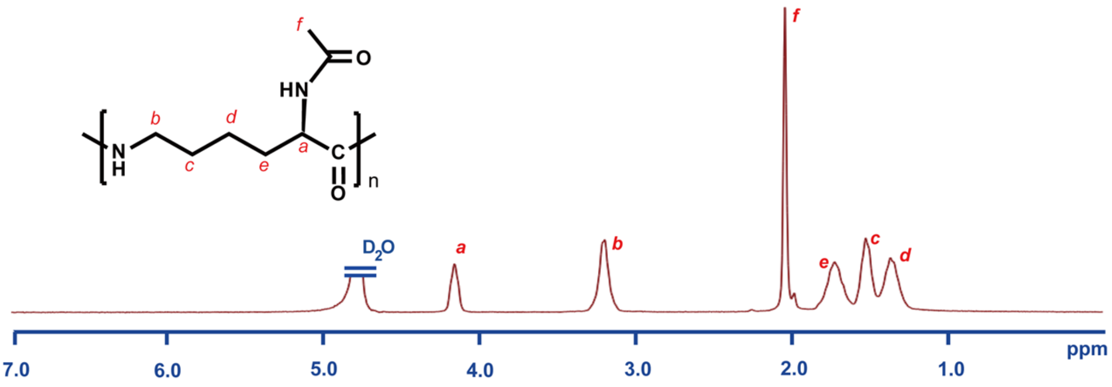

Figure S11. ^{1}H-NMR spectrum of acetylated ε-PL in D_2O

References

1. Guo, J.; Wei, Y.; Zhou, D.; Cai, P.; Jing, X.; Chen, X.-S.; Huang, Y. Biomacromolecules 2011, 12, 737.

2. Guo, L.; Wang, C.; Zhao W.; Li H.; SunW.; Shen Z. *Dalton Transactions* **2009**, 5406-5410.

3. Ho, C. H.; Odermatt, E.; Berndt, I.; Tiller, J. C. *Journal of Polymer Science Part A: Polymer Chemistry* **2009**, 46, 5053-5063.

4. Mondragón, L.; Mas, N.; Ferragud, V.; de la Torre, C.; Agostini, A.; Martínez-Máñez, R.; Sancenón, F.; Amorós, P.; Pérez-Payá, E.; Orzáez, M. *Chemistry – A European Journal* **2014**, 20, 5271.

5. Becke, A. D. *Physical Review A* **1988**, *38*, 3098.

6. Becke, A. D. *The Journal of Chemical Physics* **1992**, *96*, 2155.

7. Becke, A. D. *The Journal of Chemical Physics* **1992**, *97*, 9173.

8. Becke, A. D. *The Journal of Chemical Physics* **1993**, *98*, 5648.

9. Lee, C.; Yang, W.; Parr, R. G. *Physical Review B* **1988**, *37*, 785.

10. Miehlich, B.; Savin, A.; Stoll, H.; Preuss, H. *Chemical Physics Letters* **1989**, *157*, 200.

11. Frisch, M. J.; Trucks, G. W.; Schlegel, H. B.; Scuseria, G. E.; Robb, M. A.; Cheeseman, J. R.; Scalmani, G.; Barone, V.; Mennucci, B.; Petersson, G. A.; Nakatsuji, H.; Caricato, M.; Li, X.; Hratchian, H. P.; Izmaylov, A. F.; Bloino, J.; Zheng, G.; Sonnenberg, J. L.; Hada, M.; Ehara, M.; Toyota, K.; Fukuda, R.; Hasegawa, J.; Ishida, M.; Nakajima, T.; Honda, Y.; Kitao, O.; Nakai, H.; Vreven, T.; Montgomery Jr., J. A.; Peralta, J. E.; Ogliaro, F.; Bearpark, M. J.; Heyd, J.; Brothers, E. N.; Kudin, K. N.; Staroverov, V. N.; Kobayashi, R.; Normand, J.; Raghavachari, K.; Rendell, A. P.; Burant, J. C.; Iyengar, S. S.; Tomasi, J.; Cossi, M.; Rega, N.; Millam, N. J.; Klene, M.; Knox, J. E.; Cross, J. B.; Bakken, V.; Adamo, C.; Jaramillo, J.; Gomperts, R.; Stratmann, R. E.; Yazyev, O.; Austin, A. J.; Cammi, R.; Pomelli, C.; Ochterski, J. W.; Martin, R. L.; Morokuma, K.; Zakrzewski, V. G.; Voth, G. A.; Salvador, P.; Dannenberg, J. J.; Dapprich, S.; Daniels, A. D.; Farkas, Ö.; Foresman, J. B.; Ortiz, J. V.; Cioslowski, J.; Fox, D. J.; Gaussian, Inc.: Wallingford, CT, USA, 2009.
